# Gestational exposure to cannabidiol leads to glucose intolerance in 3-month-old male offspring

**DOI:** 10.1530/JOE-23-0173

**Published:** 2023-11-23

**Authors:** Sebastian R Vanin, Kendrick Lee, Mina Nashed, Brennan Tse, Mohammed Sarikahya, Sukham Brar, Gregg Tomy, Amica-Mariae Lucas, Thane Tomy, Steven R Laviolette, Edith J Arany, Daniel B Hardy

**Affiliations:** 1 Departments of Obstetrics and Gynaecology, and Physiology and Pharmacology, Schulich School of Medicine and Dentistry, University of Western Ontario, London, Ontario, Canada; 2 Department of Anatomy and Cell Biology, University of Western Ontario, London, Ontario, Canada; 3 Department of Pathology and Laboratory Medicine, Schulich School of Medicine and Dentistry, The Lawson Health Research Institute and Children's Health Research Institute, University of Western Ontario, London, Ontario, Canada; 4 Department of Chemistry, University of Manitoba, Winnipeg, Manitoba, Canada; 5 The Lawson Health Research Institute and the Children's Health Research Institute, London, Ontario, Canada

**Keywords:** cannabidiol, pancreas, glucose intolerance, liver, insulin signaling, endocannabinoid system

## Abstract

Reports in North America suggest that up to 20% of young women (18–24 years) use cannabis during pregnancy. This is concerning given clinical studies indicate that maternal cannabis use is associated with fetal growth restriction and dysglycemia in the offspring. Preclinical studies demonstrated that prenatal exposure to Δ9-tetrahydrocannabinol, the main psychoactive component of cannabis, in rat dams led to female-specific deficits in β-cell mass and glucose intolerance/insulin resistance. Yet to date, the contributions of cannabidiol (CBD), the primary nonpsychoactive compound in cannabis, remain elusive. This study aimed to define the effects of *in utero* cannabidiol (CBD) exposure on postnatal glucose regulation. Pregnant Wistar rat dams received daily intraperitoneal injections of either a vehicle solution or 3 mg/kg of CBD from gestational day (GD) 6 to parturition. CBD exposure did not lead to observable changes in maternal or neonatal outcomes; however, by 3 months of age male CBD-exposed offspring exhibited glucose intolerance despite no changes in pancreatic β/α-cell mass. Transcriptomic analysis on the livers of these CBD-exposed males revealed altered gene expression of circadian rhythm clock machinery, which is linked to systemic glucose intolerance. Furthermore, alterations in hepatic developmental and metabolic processes were also observed, suggesting gestational CBD exposure has a long-lasting detrimental effect on liver health throughout life. Collectively, these results indicate that exposure to CBD alone in pregnancy may be detrimental to the metabolic health of the offspring later in life.

## Introduction

Reports in North America suggest that 3–22% of women, ages 18–24, consume cannabis during pregnancy (Young-Wolff *et al.* 2019). This high rate of prenatal cannabis use is attributed to the perception that cannabis does not pose a significant risk to the health of the mother or the fetus in pregnancy (Bayrampour *et al.* 2019). This is of major concern, considering that maternal cannabis use is associated with both adverse neurodevelopmental and metabolic (i.e. dysglycemia and dyslipidemia) outcomes in the offspring (English *et al.* 1997, Conner *et al.* 2016, Gunn *et al.* 2016, Campbell *et al.* 2018, Moore *et al.* 2022). Yet to date, little is known about the effects of the major constituents of cannabis, Δ9-tetrahydrocannabinol (Δ9-THC) and cannabidiol (CBD), on postnatal glucose homeostasis. Both cannabinoids act on the receptors of the endocannabinoid system (ECS) which plays a role in the regulation of energy homeostasis, especially via its effects on glucose regulation (Matias & Di Marzo 2007). Indeed, the ECS in the pancreas is involved in the development of the endocrine islets, as well as glucose-stimulated insulin secretion (Malenczyk *et al.* 2013, 2015). Moreover, in the liver, activation of the ECS also contributes to dysglycemia (Sun *et al.* 2014).

Δ9-THC is the main psychoactive component of cannabis, responsible for the ‘high’ effect cannabis users experience. In contrast, CBD is the primary nonpsychoactive compound in cannabis, and is medicinally used for the treatment of certain forms of epilepsy, schizophrenia, and depression (Crippa *et al.* 2018). Importantly, CBD can cross the placenta and enter fetal circulation to potentially impact the development of key metabolic organs (Bailey *et al.* 1987, Kim *et al.* 2018). Our laboratory recently published that gestational exposure to Δ9-THC alone in rat dams leads to symmetrical fetal growth restriction and female-specific deficits in β-cell mass, glucose intolerance, and peripheral insulin resistance (Gillies *et al.* 2020, Natale *et al.* 2020, Asadi *et al.* 2022). However, the safety of CBD in pregnancy and its impact on glucose homeostasis in postnatal life remain largely unknown. This is of great importance considering that CBD is promoted to be safe in pregnancy (Sarrafpour *et al.* 2020). Given that (i) CBD interacts with the ECS in a similar manner as Δ9-THC, and (ii) the ECS is implicated in pancreatic development and glucose homeostasis, we postulate that maternal exposure to CBD in rat pregnancy will lead to deficits in both pancreatic β-cell mass and glucose homeostasis in the offspring.

## Research design and methods

### Animals

Pregnant Wistar rats were obtained from Charles River Company and kept under 12 h darkness:12 h light cycle at 22°C at Western University Animal House Facility. Animals had access to regular chow diet and water *ad libitum*. All animal experiments were done based on the approved Animal Use protocol by the subcommittee of Canadian Council of Animal Care, Western University (AUP# 2019-126) in accordance with the ARRIVE guidelines (https://arriveguidelines.org). All dams arrived at the animal care facilities on gestational day (GD) 3 and were given 3 days to acclimatize prior to handling. Dams were randomly assigned to either receive 3 mg/kg of CBD (Canopy, Canada) (*n* = 8) or vehicle (18:1 saline–Cremophor) (*n* = 8) via intraperitoneal injections (i.p.) from GD6 to GD22 (parturition). This route and concentration of CBD results in circulating concentrations of ~9 ng/mL (Klein *et al.* 2011), which are within the range reported in cannabis users with a half-life of ~7 h (Ignatowska-Jankowska *et al.* 2011, Deiana *et al.* 2012, Callejas *et al.* 2018). This also falls in range (10–335 ng/mL) with what has been reported in human umbilical cord samples (Kim *et al.* 2018). While i.p. is not the standard means of human cannabinoid delivery, the goal of the experimental design was to generate a serum concentration of CBD in the rat that was within the range of human use, while also considering that oral gavage can be more stressful, and edibles have poorer bioavailability and slower adsorption with food (Dinieri & Hurd 2012). In our hands, we have been very successful in our i.p. injections, ensuring we do not accidently inject fetuses/placentae and lead to any alterations in fetal demise/gestational length (Natale *et al.* 2020). Since exposure to cannabinoids impairs implantation in the rodent (Harbison & Mantilla-Plata 1972), our experiments started at gestational day 6. At birth, litters from both treatment groups were sexed based on anogenital distance and culled to *n* = 8 pups/litter (four males and four females). For each dam, gestation length, litter size, and the number of stillbirths were recorded. From these data, the live birth index ((number of live offspring/number of offspring delivered) × 100 was also determined. At birth, the offspring’s body weights, liver weights, and liver–body weight ratio were recorded and all measurements were averaged across pups in a litter. The remaining pups were followed through to postnatal day (PND) 21, and 3 months of age where they were fasted for ~14 h prior to being sacrificed via an overdose of pentobarbital (100 mg/kg i.p.). Pancreatic tissue was collected from all sacrificed animals, weighed, fixed in paraformaldehyde (4% v/v in PBS), and embedded in paraffin. Liver tissue was also collected, placed in TRIzol reagent (ThermoFisher), and flash frozen in liquid N_2_ for later RNA-Sequencing analysis. Livers were collected between 09:00 and 12:00 h. Prior to sacrifice, the 3-month-old offspring underwent i.p. glucose tolerance tests (GTTs).

### High-performance liquid chromatography–tandem mass spectrometry

Wistar PND1 rat livers were collected, flash frozen in liquid nitrogen, and stored at −80°C until sample processing. Homogenized rat livers (0.02 g) were placed into a 2.0 mL microcentrifuge tube and spiked with the following mass-labeled internal standards: 6 µL TRP-d5, KYN-d4 (each at 25 ng/µL) 10 µL of THC-d3 and CBD-d3 (each at 15 ng/µL) and 150 µL of CORT-d4 (1 ng/µL). Extractions were performed by adding 1.35 mL of 35:65 H_2_O–ACN to each tube, sonicating in an ice bath for 12 min, vortexing for 1 min and centrifuging at 21,000 ***g*** for 15 min. The supernatant was removed, and the remaining tissue was re-extracted as before. Extracts were combined and reduced in volume to 1.5 mL with a gentle stream of ultrahigh purity N_2_ prior to HPLC-MS/MS analysis.

Method detection limit for CBD, determined according to the procedure described in Euachem Guide for Method Validation (Magnusson & Örnemark) was 0.06 ng/g. The detection and quantitation of target analytes were based on previously published validated methods (Brown *et al.* 2019, Wish *et al.* 2022). In brief, for the cannabinoids, separations were achieved using an XSelect® HSS T3 C18 column (2.5 mm × 2.1 mm, 50 mm) in conjunction with a VanGuard® HSS T3 C18 cartridge (3 mm × 2.1 mm, 5 mm) and ionization in the positive mode electrospray mode. The HPLC used was an Agilent 1100 (Palo Alto, CA, USA) with a CTC PAL autosampler. The HPLC was coupled to a Sciex 365 triple quadrupole mass spectrometer retrofitted with an HSID Ionics EP+ orthogonal ionization source. Details of the multiple reaction monitoring ion transitions used for all our target analytes and mass-labeled internal standards can be found in Brown *et al.* (2019) and Wish *et al.* (2022).

### Intraperitoneal glucose tolerance test and fasting serum insulin measurement

Glucose homeostasis was assessed in 3-month-old offspring via an i.p. GTT, as previously described (Gillies *et al.* 2020). Briefly, five to eight offspring per treatment group per sex (*n* = 6 vehicle-exposed males from four litters, *n* = 8 CBD-exposed (3 mg/kg) males from 5 litters, *n* = 5 vehicle-exposed females, *n* = 6 CBD-exposed (3 mg/kg) females) were fasted for 12 h overnight. Animals were administered 2 g/kg glucose via i.p. injection, and blood glucose levels measured with a OneTouch Ultra2 handheld glucometer (LifeScan, Zug, Switzerland) at 0, 5, 15, 30, 60, 90, and 120 min post injection. Total glucose response to the bolus injection was measured by calculating the area under the curve (AUC). Fasting glucose levels and fasting serum insulin levels were collected and quantified using an ELISA kit (Crystal Chem, Elk Grove Village, IL, USA). HOMA1-IR and HOMA1-B were also calculated (Wallace *et al.* 2004).

### Immunohistochemistry and endocrine pancreas morphometry

Paraffin-embedded pancreata of PND21 (*n* = 3 vehicle-exposed and CBD-exposed males from unique litters) and 3-month-old (*n* = 4 vehicle-exposed and CBD-exposed males from unique litters) offspring were cut into 5 µm sections and mounted on SuperFrost Plus glass slides (Fisher Scientific) as previously described (Gillies *et al.* 2020). Tissues were deparaffinized in xylene, rehydrated in an ethanol series (100%, 90%, 70%), and permeabilized using 0.3% Triton-X. Slides were incubated with rabbit anti-glucagon IgG (1:750; Novus Biologicals, Centennial, CO, USA) and mouse anti-insulin IgG (1:2000; Sigma-Aldrich). Slides were then incubated with donkey anti-mouse 488 (1:500) and anti-rabbit 647 (1:500) fluorescent secondary antibodies (Thermofisher, Toronto, ON, Canada), and DAPI (ThermoFisher) to counterstain nuclei. Analysis of sections was performed as previously described (Gillies *et al.* 2020). Whole pancreatic tissue sections were microphotographed with a 2.5× objective lens, and a composite image obtained using Microsoft Image Composite Editor (Version 2.0.3.0, 2015). The mean islet density was calculated by counting the total number of islets in each pancreatic section and dividing by the total pancreatic section area. Fractional β- and α-cell areas were calculated by dividing the insulin- and glucagon-positive areas, respectively, by the total pancreatic tissue area. β- and α-cell mass were calculated by multiplying the fractional β- and α-cell area by the pancreatic weight. Average % β- and α-cell area per islet area was calculated by dividing the insulin- and glucagon-positive areas, respectively, by the total islet area. All calculations were averaged across three tissue sections per animal for PND21 pancreas and two tissue sections per animal for 3-month pancreas.

### Bulk liver RNA-sequencing (RNA-Seq)

Previously collected and flash frozen livers from 3-month-old male vehicle-exposed (*n* = 6 from 3 litters) and CBD-exposed (*n* = 5 from 4 litters) offspring were sent to Genome Quebec (Montreal, Quebec, Canada) for total RNA extraction, library preparation, and RNA-Seq. RNA quality was assessed using the RNA integrity number (RIN). All RIN scores were ≥ 7.0. Paired end reads (25 million, 100 bp) were sequenced on the Illumina NovaSeq platform. Raw reads were aligned and annotated with the latest ENSEMBL rat genome release available (mRatBN7.2) using the R package Rsubread with default settings (Liao *et al.* 2019). Raw counts were generated using the Rsubread sub package featureCounts. Lowly expressed genes were filtered out using a counts per million (CPM) cutoff of 0.4 in at least two or more samples. Based on principal component analysis (PCA), two CBD liver samples were removed as outliers from further analysis (Supplementary Fig. 1, see section on supplementary materials given at the end of this article). Normalization and differential expression (DE) analysis were done using the edgeR R package (Chen *et al.* 2017). Briefly, counts were normalized using the trimmed means of M-values (TMM) method. DE was determined using the exact test method. To account for multiple testing, *P*-values were adjusted using Benjamini–Hochberg false discovery rate (FDR) correction. An FDR cutoff of <0.05 was used to determine significance.

The gprofiler2 (Kolberg *et al.* 2020) R interface for g:Profiler (version e109_eg56_p17_1d3191d) was used to convert ENSEMBL gene IDs to gene symbols, and to perform functional enrichment analysis on the DE genes from contrasts of interest, with a g:SCS threshold of 0.05 (Raudvere *et al.* 2019). The databases included in the analysis were the Gene Ontology (GO) database, the Kyoto Encyclopedia of Genes and Genomes (KEGG) database, the Reactome (REAC) database, the TRANSFAC (TF) database, and the WikiPathways (WP) database. There are also three GO sub-categories: biological process (BP), cellular component (CC), and molecular function (MF). To examine the expression profiles of genes within pathways of interest, we generated a matrix of the log_2_ CPM values of genes in those pathways, and generated heatmaps using the pheatmap R package (Hu 2021). Values across rows (genes) were centralized and scaled for comparison, and Ward’s clustering algorithm (ward.D2) used for hierarchical clustering.

Given the high number of repetitive terms found in the functional enrichment analysis results for the GO:BP database, we next aimed to cluster the results based on likeness. Specifically, we calculated the Jaccard similarity coefficient for all pairwise comparisons of GO:BP terms based on the genes within each term. The resulting similarity matrix of the top 50 GO:BP terms, ranked by adjusted *P*-value, were visualized using the pheatmap package. Ward’s clustering algorithm was again used for hierarchical clustering.

### Protein extraction and western blot

Flash frozen livers from 3-month-old male vehicle-exposed (*n* = 7 from 3 litters) and CBD-exposed (*n* = 6 from 4 litters) rats had proteins extracted as previously described (Oke *et al.* 2020). Samples were loaded into 4–12% Bis-Tris gels (Invitrogen) at 20 µg per well and separated by gel electrophoresis. Proteins were then transferred onto a PVDF membrane (Thermo Scientific) at 60 V for 2 h, followed by blocking overnight at 4⁰C in 1× Tris-buffered saline/Tween 20 (TBST) buffer with 5% nonfat milk. Blocked blots were probed with primary antibodies (Table 1) diluted in the blocking solution for 1 h at room temperature, followed by probing with the appropriate secondary antibody (Table 1) for 1 h at room temperature. Protein bands were visualized via chemiluminescence using Bio-Rad Clarity Max Western ECL Substrate solution (Bio-Rad Laboratories Canada Ltd.), and imaged using a Bio-Rad ChemiDoc XRS+ Imaging System. Membranes were also stained with Ponceau S stain (0.1% (w/v) Ponceau S in 5% (v/v) acetic acid) to detect total protein content. Band intensities were normalized to total protein content.

**Table 1:** Western blot primary and secondary antibodies, dilutions, and company information.

Antibody name	Source	Dilution	Company
AKT2	Rabbit	1:1000	Cell Signaling Technology Inc. (3063S)
PI3K p85α	Rabbit	1:1000	Cell Signaling Technology Inc. (4257P)
SAPK/JNK	Rabbit	1:1000	Cell Signaling Technology Inc. (9252S)
p44/42 MAPK	Rabbit	1:1000	Cell Signaling Technology Inc. (9102S)
mTOR	Rabbit	1:1000	Cell Signaling Technology Inc. (2972S)
GLUT2	Mouse	1:1000	Santa Cruz Biotechnology Inc. (sc-30081)
ALT	Rabbit	1:1000	Abcam Inc. (ab236658)
Horse anti-rabbit IgG, HRP-linked		1:10,1000	Cell Signaling Technology Inc. (7074P2)
Horse anti-mouse IgG, HRP-linked		1:10,1000	Cell Signaling Technology Inc. (7076s)

### Statistical analysis

Statistical analysis was performed using R software (version 4.2.3) or GraphPad (version 9.5.1) using a Student’s unpaired *t*-test or a Welch’s two-sample *t*-test where necessary. Outliers were determined using Grubb’s test. Results are expressed as means ± s.e.m., unless otherwise stated. The threshold for significance was set as *P* < 0.05.

## Results

### Prenatal CBD exposure in the rat does not affect maternal or neonatal outcomes

Prenatal CBD exposure did not lead to any changes in the measured maternal or neonatal outcomes, including gestational length, maternal food intake, pregnancy weight gain, litter size, and pup survival to PND4 (Table 2). Additionally, we did not observe any changes to body weight, liver weight, and liver–body weight ratio at birth. Utilizing HPLC, we also measured CBD concentrations in the livers of PND1 pups to confirm that CBD was entering into fetal circulation and tissues. Indeed, CBD was successfully detected in the livers (~40 ng/g) of CBD-exposed offspring (Table 2).

**Table 2:** Maternal and neonatal outcome measurements (*n* = 3–8 fetuses/litter from 5 to 6 dams/treatment), and PND1 pup liver CBD concentration measurements. Significant differences in values were assessed by a Student’s unpaired *t*-test (*P* < 0.05).

Maternal/neonatal outcome measures	Vehicle	CBD 3 mg/kg	*P*
Gestational length (days)	21.92 ± 0.08	21.86 ± 0.10	0.880
Average food intake: days 12–14 (g/day)	24.75 ± 0.99	24.75 ± 1.059	0.595
Average food intake: days 18–20 (g/day)	24.00 ± 0.62	25.85 ± 1.03	0.365
Pregnancy weight gain: GD6–GD21 (g)	105.0 ± 4.38	114.5 ± 4.54	0.428
Litter size (*n*)	9.33 ± 0.50	10.00 ± 0.59	0.739
Survival to PND4 (%)	100	100	1
PND1 offspring body weight (g)	6.19 ± 0.10	6.71 ± 0.19	0.143
PND1 offspring liver weight (g)	0.30 ± 0.01	0.31 ± 0.02	0.838
PND1 offspring liver–body weight ratio	0.0497 ± 0.0001	0.0462 ± 0.0020	0.398
PND1 liver CBD concentration (ng/g of liver)	1.0 ± 5.7	41.9 ± 24.0	

### Prenatal CBD exposure in the rat results in sex-specific glucose intolerance at 3 months of age

**Figure 1:**
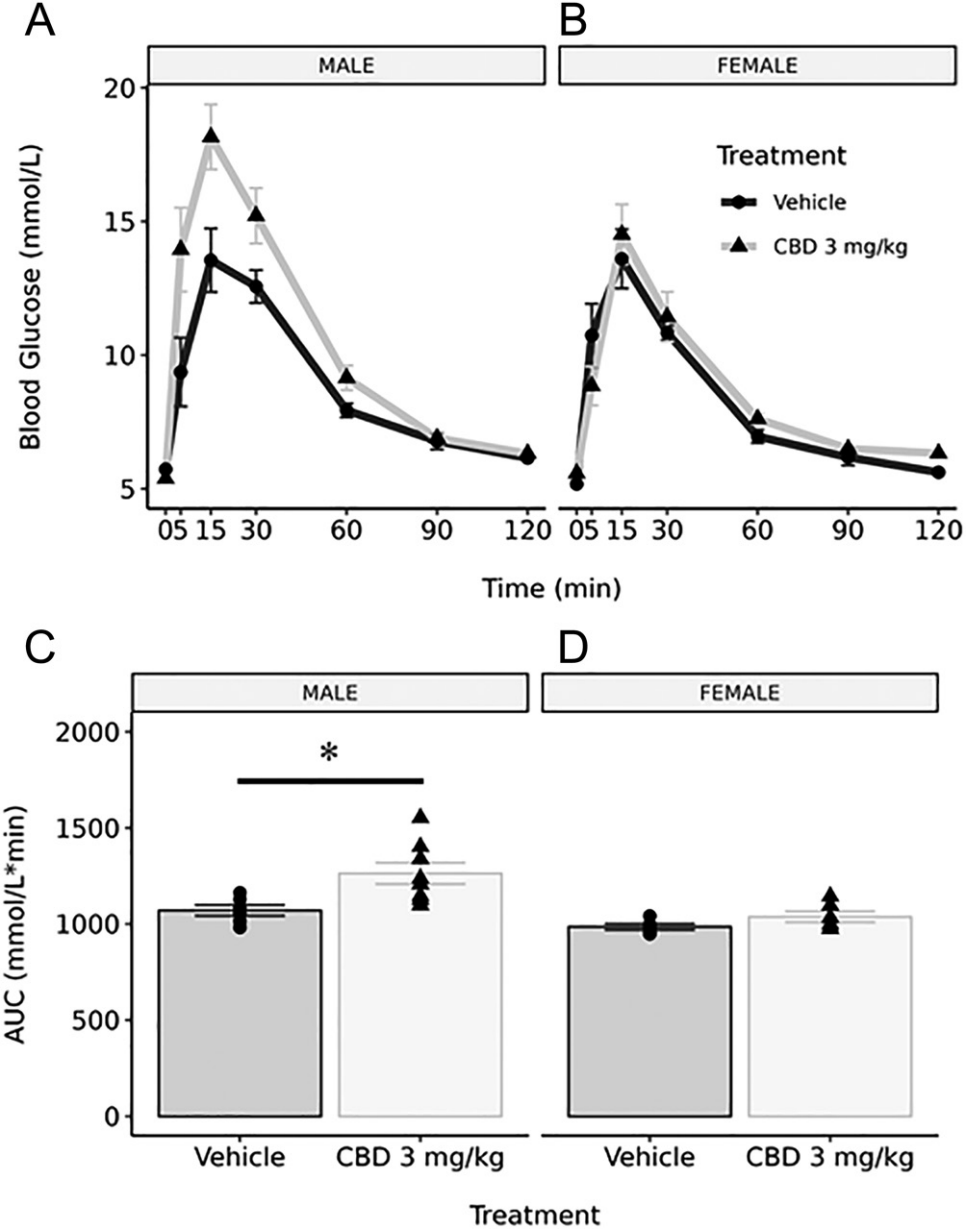
Gestational exposure to 3 mg/kg of CBD leads to glucose intolerance in male Wistar rat offspring at 3 months of age. Blood glucose levels were measured at 0, 5, 15, 30, 60, 90, and 120 min following a 2 g/kg glucose challenge in 3-month-old male (A; *n* = 6 vehicle, *n* = 8 CBD 3 mg/kg)) and female (B; *n* = 5 vehicle, *n* = 6 CBD 3 mg/kg) offspring. The resulting glucose response curves had integrated area under the curve (AUC; ± s.e.m.) calculated for males (C) and females (D). CBD-exposed (3 mg/kg) males had a significantly increased AUC value when compared to vehicle-exposed counterparts. There were no significant changes in AUC values between CBD-exposed and vehicle-exposed females. Significant differences in AUC values were assessed by a Student’s unpaired* t*-test (*, *P* < 0.05).

To determine the impact of gestational CBD exposure on the regulation of blood glucose in postnatal life, i.p. GTTs were performed on 3-month-old CBD- and vehicle-exposed offspring. For 3-month male offspring, overall integrated AUC for blood glucose was significantly elevated in CBD-exposed males (1264 ± 55.40 mmol/L min^−1^ for CBD, 1070 ± 27.83 mmol/L min^−1^ for vehicle, *P* = 0.0158; Fig. 1A and C). However, this was not associated with any changes in fasting serum insulin, HOMA-IR, or HOMA-B scores (Table 3). In contrast, female CBD-exposed offspring did not exhibit any changes in integrated AUC when compared to their vehicle-exposed counterparts (Fig. 1B and D). Additionally, there were no changes in fasting insulin levels, or HOMA-IR and HOMA-B scores in CBD-exposed female offspring (Table 3).

**Table 3:** Fasting Insulin (ng/mL), fasting glucose (mmol/L), HOMA-IR, and HOMA-B values for 3-month-old vehicle and CBD-exposed males (A) and females (B). Fasting blood samples were collected for vehicle-exposed (*n* = 5 animals per sex) and CBD-exposed (3 mg/kg) (*n* = 5 females, *n* = 6 males) 3-month-old male and female Wistar rat offspring. Fasting glucose (mmol/L) levels were measured in all samples using a glucometer. Fasting insulin (ng/mL) levels were determined via an insulin ELISA. Indices of insulin resistance (HOMA-IR and QUICKI) and β-cell function (HOMA-B) were calculated using fasting glucose and insulin levels. There were no significant changes in fasting insulin or glucose levels between treatments for either sex, or for the indices shown. Significant differences in were assessed via a Student’s *t*-test (*, *P* < 0.05). All values are means ± s.d.

Sex	Measurement	Vehicle	CBD (3 mg/kg)	*P*
Male	Fasting insulin (ng/mL)	0.22 ± 0.06	0.42 ± 0.30	0.170
	Fasting glucose (mmol/L)	5.60 ± 0.47	5.32 ± 0.56	0.389
	HOMA-IR	1.39 ± 0.43	2.45 ± 1.71	0.197
	HOMA-B	54.41 ± 13.72	123.1 ± 90.01	0.121
Female	Fasting insulin (ng/mL)	0.34 ± 0.23	0.22 ± 0.09	0.307
	Fasting glucose (mmol/L)	5.3 ± 0.31	5.82 ± 0.40	0.051
	HOMA-IR	2.02 ± 1.39	1.44 ± 0.67	0.429
	HOMA-B	94.86 ± 55.89	45.97 ± 12.39	0.122

### Prenatal CBD exposure in the rat does not impact male pancreatic development in postnatal life

**Figure 2:**
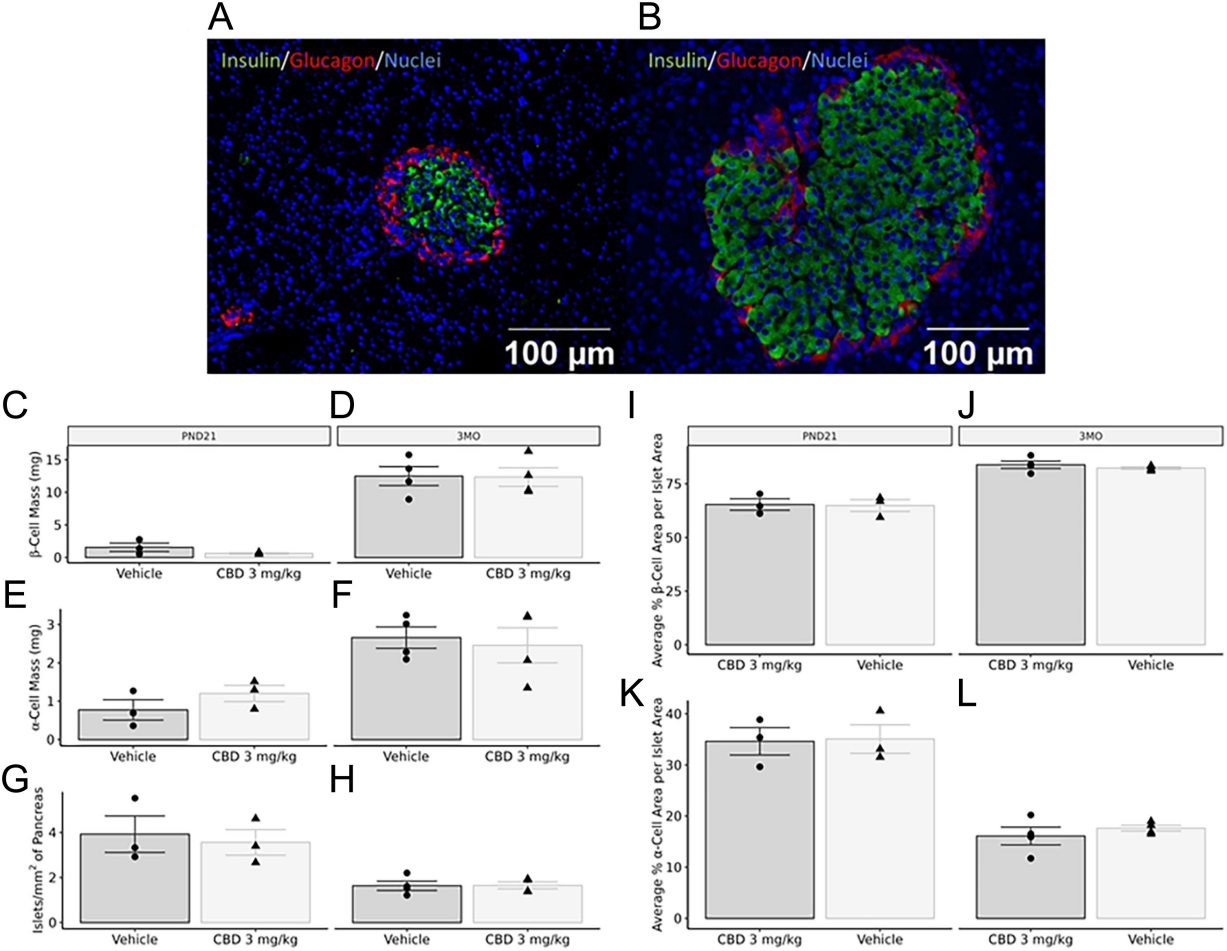
Representative immunohistochemistry of PND21 (A) and 3-month-old (B) islets immunostained for insulin, glucagon, and DAPI. Exposure to 3 mg/kg of CBD during gestation did not lead to any changes in β-cell mass, α-cell mass, or islet density in PND21 (C, D, and E) or 3-month-old (F, G, and H) male offspring pancreata. There were similarly no changes in the average % β- and α-cell area per islet area at either time point in male offspring (I–L). Values are means ± s.e.m.(*n* = 3–4). Significant differences were assessed via a Student’s *t*-test (*, *P* < 0.05).

Given the observed glucose intolerance was *exclusively* in CBD-exposed male offspring, we examined the morphology of the endocrine pancreas islets at both 3 weeks and 3 months in male offspring only. At either 3 weeks (Fig. 2C, E, and G) or 3 months (Fig. 2D, F, and H), there were no changes in β-cell mass, α-cell mass, or total islet density in male offspring. There were similarly no changes in the average % β- and α-cell area per islet area at either time point in male offspring (Fig. 2I, J, K, and L).

### Liver bulk RNA-Seq analysis

**Figure 3:**
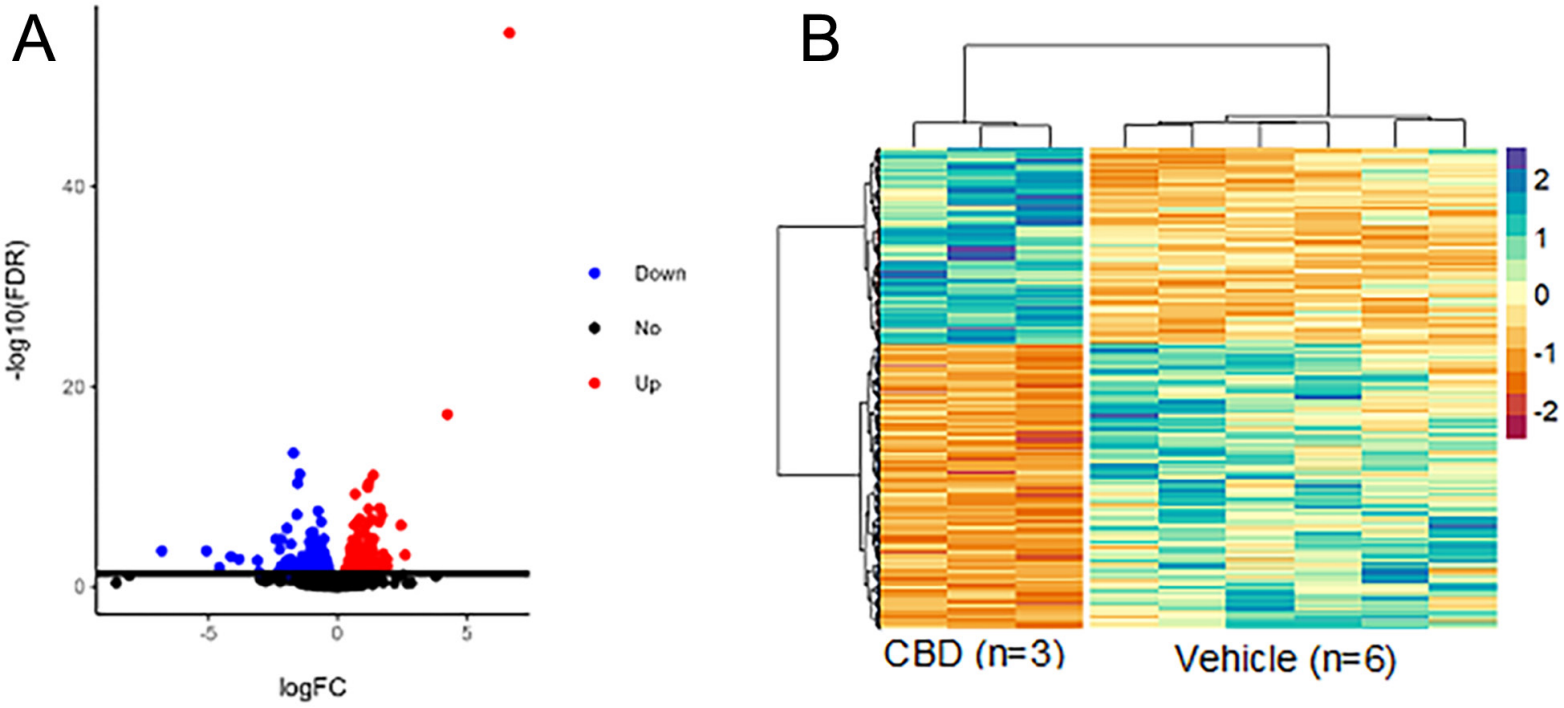
Volcano plot (A) and heatmap (B) of DE genes between livers of 3-month-old CBD-exposed and vehicle-exposed male offspring. RNA-Seq was performed on livers from 3-month-old male vehicle-exposed (*n* = 6) and CBD-exposed (*n* = 3) offspring. Using an FDR < 0.05 to define significance, there were 191 upregulated genes, and 277 downregulated genes. A) The log_2_ of the fold change plotted against the log_10_ of the FDR for all genes in the DE comparison. B) Heatmap created using the log_2_ normalized CPM values for all DE genes (rows) and samples (columns). Values were centered and scaled across rows for comparison, and clustered using Ward’s clustering algorithm.

**Figure 4:**
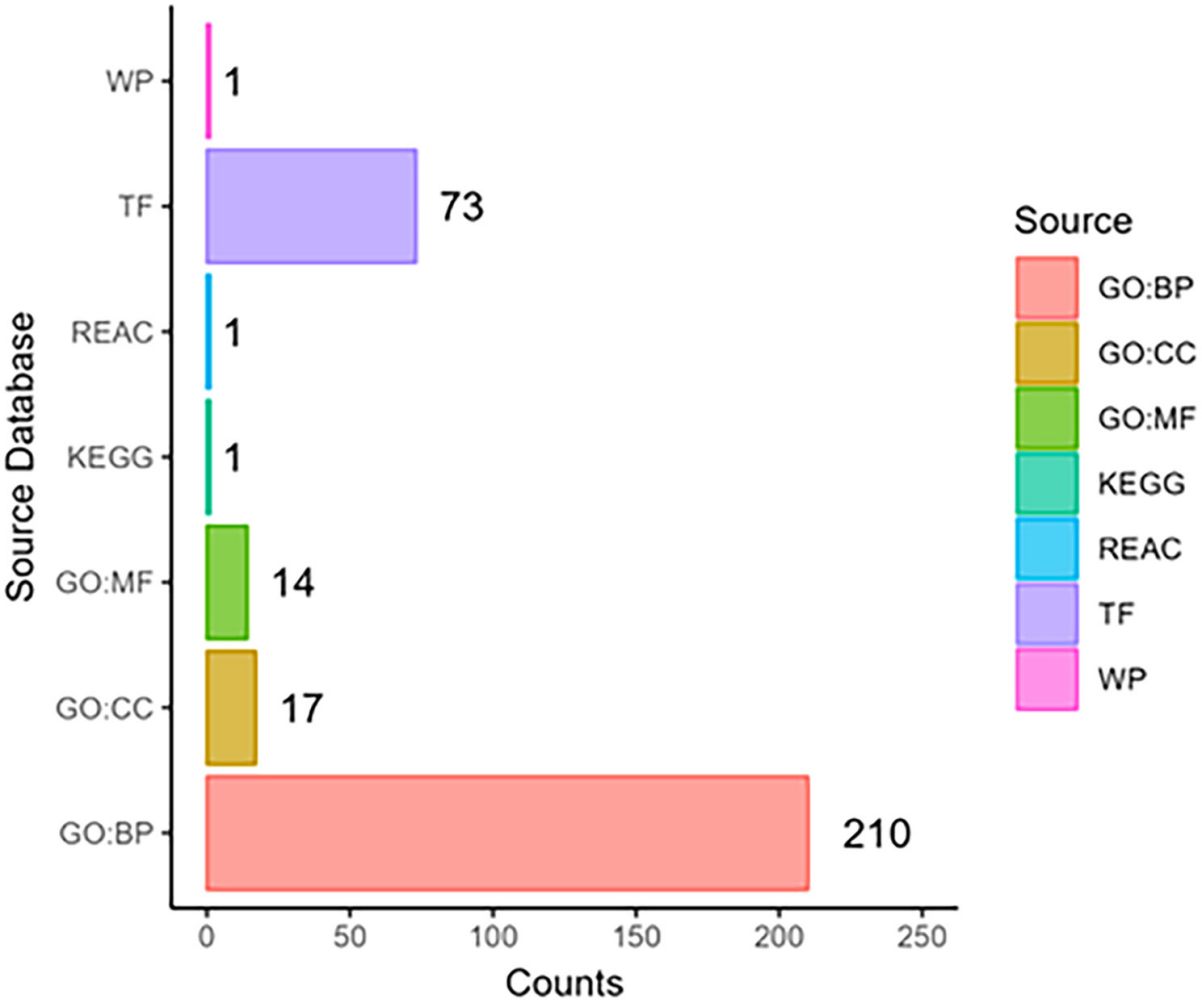
Barplot of counts of enriched terms from g:Profiler functional enrichment analysis. Functional enrichment analysis was carried out using g:Profiler (version e109_eg56_p17_1d3191d) with a g:SCS threshold of 0.05. Analysis was performed across the Gene Ontology (GO), Kyoto Encyclopedia of Genes and Genomes (KEGG), Reactome (REAC), TRANSFAC (TF), and WikiPathways (WP) databases.

**Figure 5:**
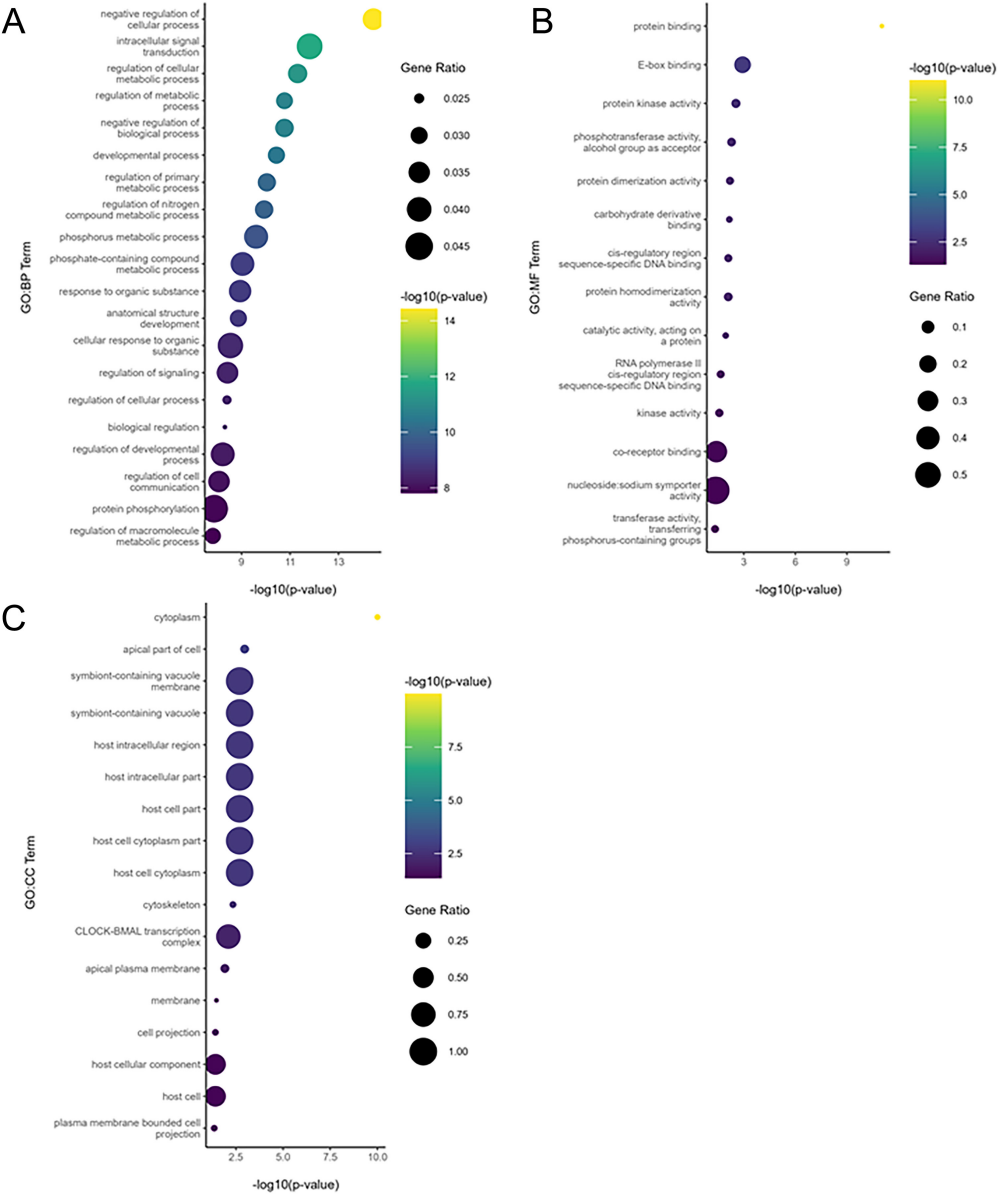
Dot Plot of The Top Enriched A) GO:BP, B) GO:CC, and C) GO:MF Terms From Functional Enrichment Analysis. Functional enrichment analysis was carried out using g:Profiler (version e109_eg56_p17_1d3191d) with a g:SCS threshold of 0.05. Only the top 20 enriched terms are shown at maximum. Each bubble is the -log_10_(*P*-value) for the respective term and is sized according to the gene ratio.

With no changes in resting glucose and pancreatic morphology in CBD-exposed offspring, we next examined if alterations in peripheral insulin-target organs might account for the glucose intolerance observed. Given the fact that the liver plays a major role in glucose metabolism and regulation (Han *et al.* 2016), we examined if there were any transcriptomic changes in the liver of CBD-exposed 3-month male offspring which could define the hepatic contribution to the observed systemic glucose intolerance. Using an FDR < 0.05 to define significance, DE analysis revealed 191 significantly upregulated genes and 277 significantly downregulated genes (Fig. 3A and B; Supplementary Table 1). Functional enrichment analysis found 264 significantly enriched terms across five different pathway databases (Fig. 4). Moreover, there were 210 enriched GO:BP terms, 17 enriched GO:CC terms, and 14 enriched GO:MF terms (Fig. 5A, B and C). In addition, there was also 1 enriched KEGG term, 1 enriched REAC term, 73 enriched TF terms, and 1 enriched WP term (Supplementary Table 2).

**Figure 6:**
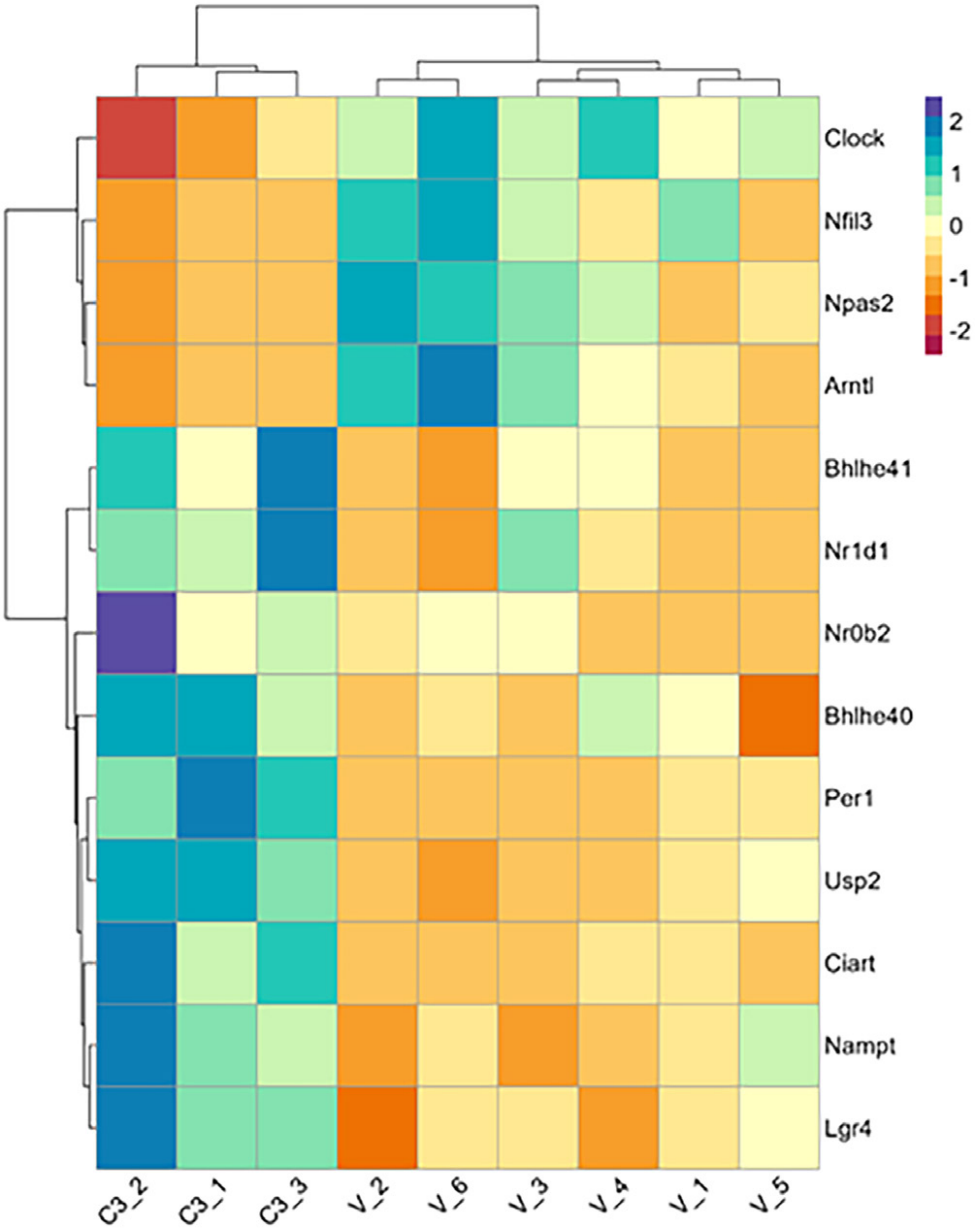
Heatmap of Circadian Rhythm Genes from Functional Enrichment Analysis. Functional enrichment analysis was carried out using g:Profiler (version e109_eg56_p17_1d3191d) with a g:SCS threshold of 0.05. Across the GO:BP, GO:CC, and KEGG circadian rhythm related terms we found 13 unique genes. The heatmap was created using the log_2_ normalized CPM values for all genes (rows) and samples (columns). Values were centered and scaled across rows for comparison, and clustered using Ward’s clustering algorithm.

Across the GO:BP, GO:CC, and KEGG enriched terms, we observed common terms related to circadian rhythm: ‘circadian regulation of gene expression’ (GO:BP), ‘CLOCK-BMAL transcription complex’ (GO:CC), and ‘Circadian rhythm’ (KEGG). We found 13 unique genes across the three circadian rhythm terms (Fig. 6). Of these 13 genes, 4 were downregulated (Clock, Nfil3, Npas2, and Arntl), and 9 were upregulated (Bhlhe41, Nr1d1, Nr0b2, Bhlhe40, Per1, Usp2, Ciart, Nampt, and Lgr4) in the livers of CBD-exposed male offspring.

**Figure 7:**
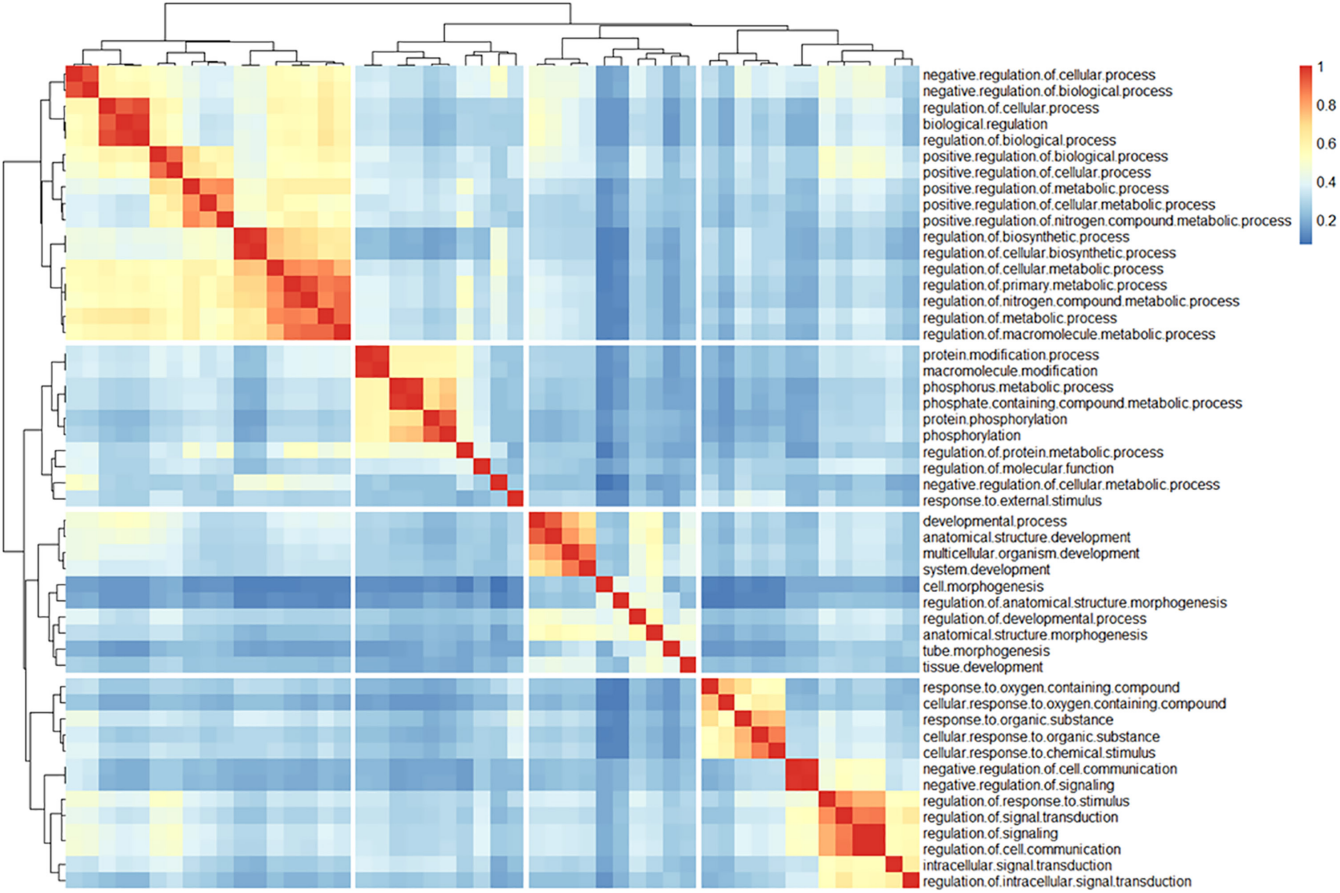
Heatmap of Similarity Matrix of Top 50 GO:BP Terms From Functional Enrichment Analysis. Functional enrichment analysis was carried out using g:Profiler (version e109_eg56_p17_1d3191d) with a g:SCS threshold of 0.05. The Jaccard similarity coefficient was calculated for all pairwise comparisons of GO:BP terms based on the genes within each term. The resulting similarity matrix of the top 50 GO:BP terms, ranked by adjusted *P*-value, were visualized using the pheatmap R package. Ward’s clustering algorithm was used for hierarchical clustering.

**Figure 8:**
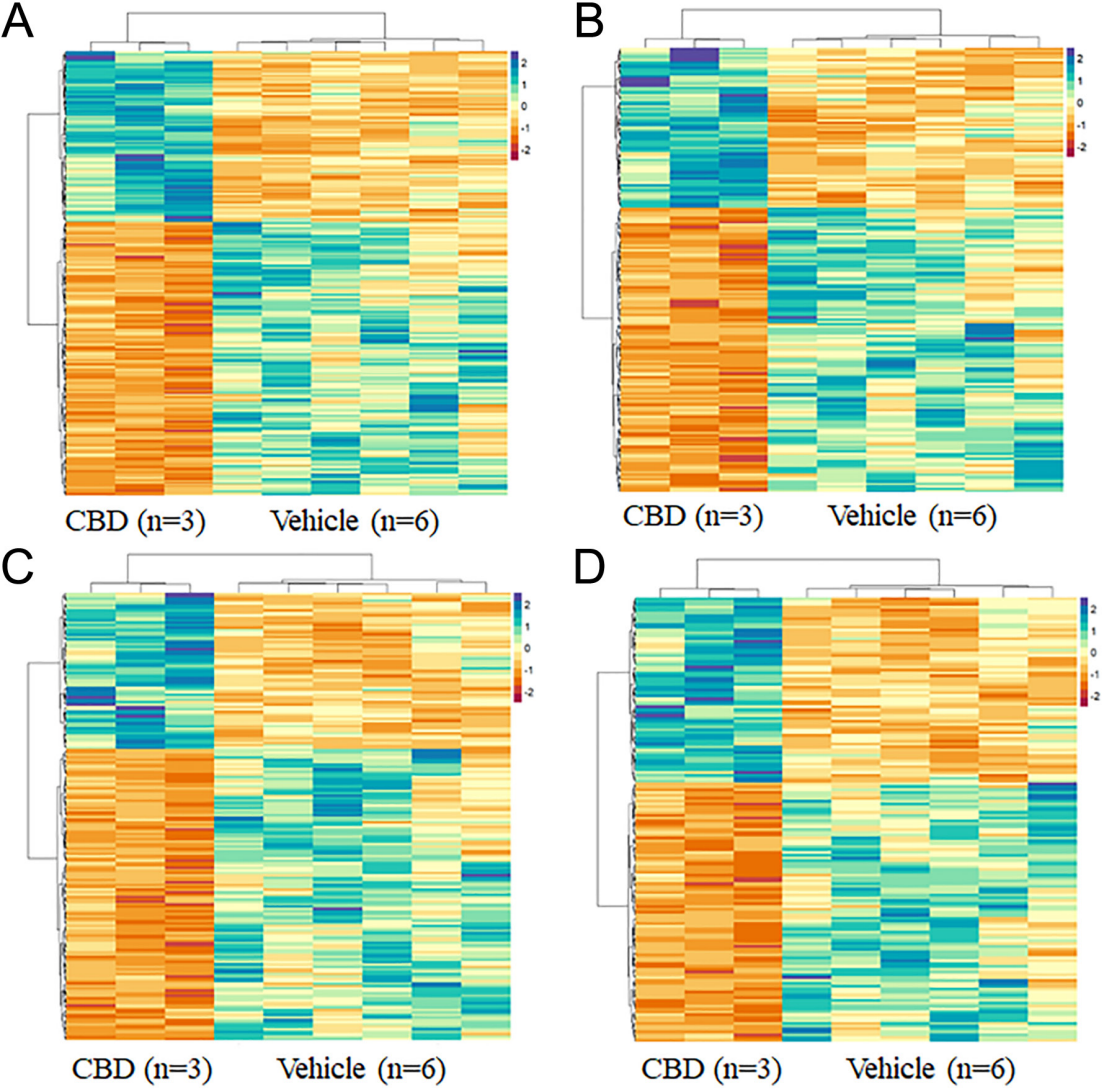
Heatmap Of DE Genes from Clustering Analysis. A) Regulation Of Cellular and Metabolic, B) Cellular Signaling and Response to Stimuli, C) Developmental and Morphogenic Processes, D) Protein Phosphorylation and Phosphorous/Phosphate Containing Compound Metabolism. Functional enrichment analysis was carried out using g:Profiler (version e109_eg56_p17_1d3191d) with a g:SCS threshold of 0.05. Clustering of the top 50 enriched GO:BP terms ranked by adjusted *P*-value found four distinct clusters of terms. The log_2_ normalized CPM values for all DE genes found within the enriched terms for each respective cluster were visualized using the pheatmap R package. Values were centered and scaled across rows for comparison, and clustered using Ward’s clustering algorithm.

Clustering of the top 50 GO:BP terms ranked by adjusted *P*-value found four distinct clusters of terms (Fig. 7): 1) regulation of cellular and metabolic processes (i.e. regulation of metabolic process, regulation of biological process, regulation of macromolecule metabolic process; Fig. 8A), 2) cellular signaling and response to stimuli (i.e., intracellular signal transduction, regulation of response to stimulus, regulation of signaling; Fig. 8B), 3) developmental and morphogenic processes (i.e. developmental process, tissue development, anatomical structure morphogenesis; Fig. 8C), and 4) protein phosphorylation and phosphorous/phosphate containing compound metabolism (i.e., phosphorous metabolic process, protein phosphorylation, phosphate-containing compound metabolic process; Fig. 8D). Using heatmaps generated with all DE genes belonging to the enriched terms in the foregoing four clusters (Fig. 8), we observed that most genes are being downregulated in the CBD-exposed offspring.

### Hepatic protein markers of insulin resistance

**Figure 9:**
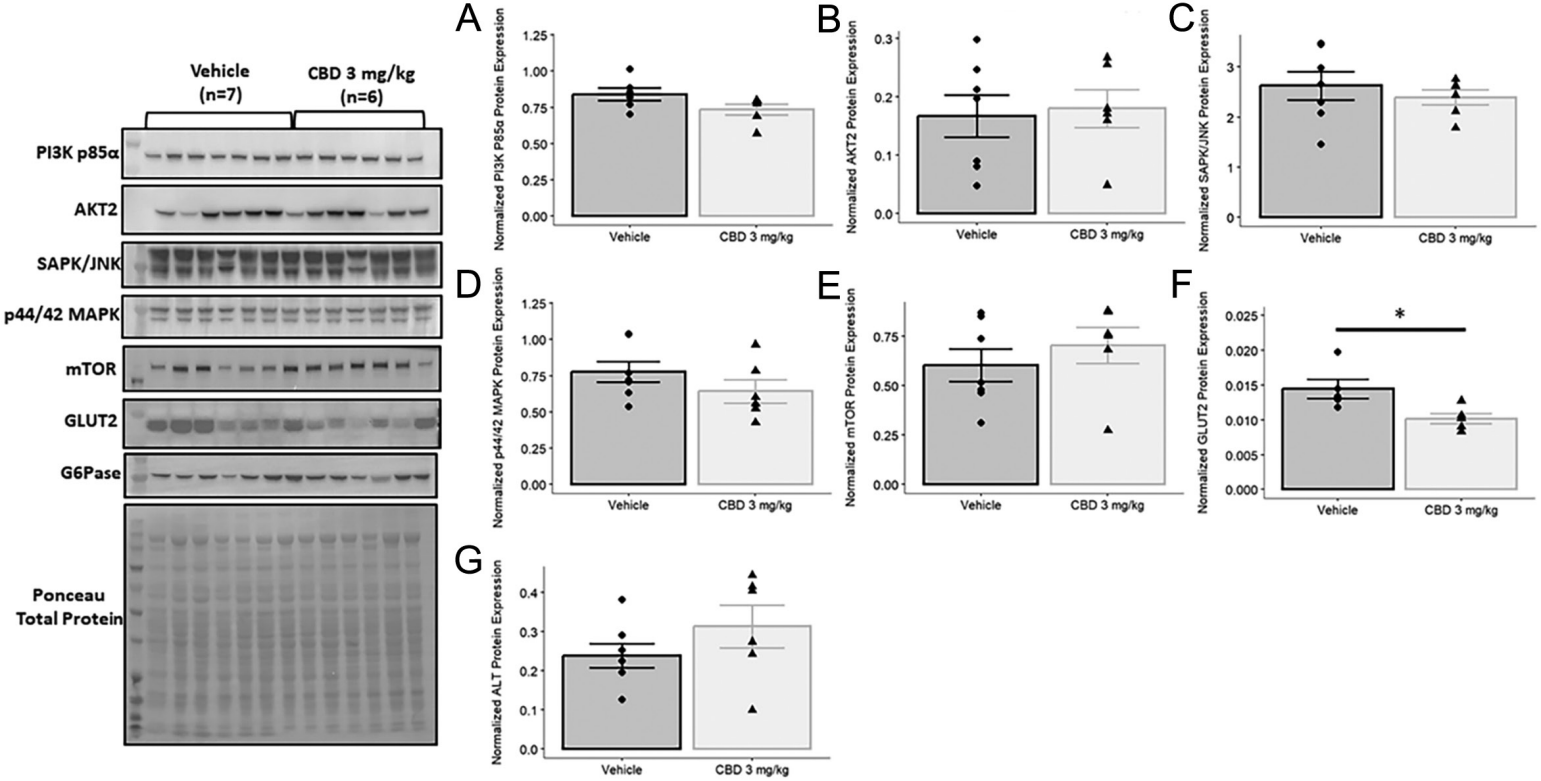
Gestational exposure to 3 mg/kg of CBD leads to reduced hepatic GLUT2 protein expression in 3-month-old male offspring. Basal protein expression levels were measured for key insulin signaling intermediaries: A) PI3K p85, B) AKT2, C) SAPK/JNK, D) p44/42 MAPK, and E) mTOR, for the principal hepatic glucose transporter GLUT2 (F), and ALT (G). Protein band intensities were normalized to total protein levels obtained via Ponceau staining. Values are means ± s.e.m.Significant differences in protein expression values were assessed by a Student’s unpaired *t*-test, or a Welch’s two sample t-test where necessary (*, *P* < 0.05).

Given the transcriptome analysis suggests that alterations in hepatic development, metabolism, and circadian rhythm may contribute to the overall glucose intolerance observed, we next examined if peripheral insulin resistance might be occurring in the CBD-exposed liver. To address this, we measured protein levels of key markers of hepatic insulin resistance at 3 months of age. Specifically, we measured the basal protein levels of key insulin signaling intermediaries (AKT2, PI3K-p85α, mTOR, SAPK/JNK), the principal hepatic glucose transporter GLUT2, and aminotransferase (ALT). CBD-exposed male offspring exhibited no changes to the fasting protein levels of insulin signaling intermediaries (Fig. 9A, B, C, D, and E), ALT (Fig. 9G) however a decrease in GLUT2 (Fig. 9F; *P* < 0.05) was observed.

## Discussion

In this study we have demonstrated that gestational exposure to 3 mg/kg of CBD i.p. alone results in glucose intolerance by 3 months of age, specifically in male offspring. Importantly, this occurred without deficits in maternal outcomes during pregnancy, or neonatal outcomes. Additionally, the observed glucose intolerance was not associated with alterations in the morphometry of the endocrine pancreas or to fasting glucose or insulin levels. This suggests that deficits in glucose regulation in peripheral insulin sensitive organs may underlie the glucose intolerance observed. As previously mentioned, the liver, an insulin sensitive organ, plays a major role in glucose metabolism and homeostasis (Han *et al.* 2016). Indeed, RNA-Seq analysis on the livers of CBD-exposed 3-month-old males revealed altered expression of genes involved in various metabolic processes, including circadian clock genes which are known to regulate glucose, lipid, and cholesterol metabolism (Reinke & Asher 2016, Shi *et al.* 2019). Additionally, there were also detrimental changes to the expression levels of genes involved in development, indicating that CBD exposure *in utero* can have long-lasting implications on postnatal liver health. Overall, this preclinical study is the first to indicate that the use of CBD in pregnancy may not be safe for overall offspring metabolic health. This is of great interest considering that The Healthy Start Study recently demonstrated that the children of mothers who used cannabis in pregnancy exhibited dysglycemia as early as 5 years of age, even after controlling for socioeconomic status, ethnicity, tobacco use, and breastfeeding (Moore *et al.* 2022). While cannabis is composed of several constituents, the current study indicates the dysglycemia observed could result from, in part, gestational CBD exposure.

Given that the ECS is known to impact hepatic development, glucose metabolism, and lipid metabolism (Sun *et al.* 2014, Liu *et al.* 2016, Chen *et al.* 2017, Bazwinsky-Wutschke *et al.* 2019) we postulated that CBD exposure would result in glucose intolerance in adult life. As previously mentioned, gestational exposure to 3 mg/kg of CBD alone did not lead to changes in maternal food intake and weight gain, or pup survival. As such, our results are not confounded by the effects of altered maternal nutrition or litter size. Using HPLC-MS/MS, we confirmed that CBD entered fetal circulation and was detectable within the livers of newborn pups. This suggests that CBD could also directly affect developmental processes involved with normal fetal organ development, including the pancreas and liver.

Interestingly, although both male and female offspring were exposed to CBD in fetal life, only males were glucose intolerant at 3-months. Specifically, CBD-exposed males demonstrated a significantly increased AUC of their glucose response curves, without any changes to fasting glucose or insulin levels. The sex-specific difference in glucose intolerance could be attributed, in part, to the effects of sex hormones on glucose homeostasis. Studies indicate that estrogen plays a protective effect against metabolic dysfunction, metabolic syndrome, and in the etiology of diabetes in pancreatic β-cells, the liver, skeletal muscle, and hypothalamus (Mauvais-Jarvis *et al.* 2013, Mauvais-Jarvis 2018). Collectively, this suggests that the female CBD-exposed offspring may be protected from developing glucose intolerance via estrogen-mediated effects, however further studies are required to elucidate the specific mechanism underlying this sexual dimorphism. Since Δ9-THC-exposed offspring exhibited sex-specific glucose intolerance despite no differences in circulating sex steroid hormones (Gillies *et al.* 2020), it also remains possible that prenatal cannabinoids may also be acting through epigenetic mechanisms (e.g. imprinting). Given the female CBD-exposed offspring were not glucose intolerant, we next investigated what mechanisms underlie the glucose intolerance observed exclusively in the CBD-exposed male offspring.

Previous studies have indicated that the ECS can regulate endocrine pancreas development, function, and proliferation (Kim *et al.* 2012, Malenczyk *et al.* 2013, 2015). Moreover, knowing that gestational exposure to Δ9-THC led to deficits in β-cell mass in postnatal life (Gillies *et al.* 2020), we investigated if gestational CBD exposure led to any deficits in endocrine pancreas development. Interestingly, we did not observe any changes to β-cell mass, α-cell mass, or total islet density in either 3-week or 3-month-old CBD-exposed male offspring. Coupled with the fact that fasting insulin and glucose levels were not significantly altered in CBD-exposed males, we postulated that the glucose intolerance is likely not due to deficits in endocrine pancreas development. This is supported by the fact that in other models of gestational insult (*e.g.* maternal exposure to the selective serotonin reuptake inhibitor fluoxetine), glucose homeostasis is also impaired without changes to β-cell mass (De Long *et al.* 2015). However, we acknowledge that a limitation to the study is that we only measured fasting insulin levels as opposed to insulin levels throughout the ipGTT. As such it is possible that while β-cell mass did not change, β-cell function, measured by insulin secretion following glucose uptake, could be impacted. While measurements of key insulin signaling intermediaries (AKT2, PI3K-p85α, mTOR, p44/42 MAPK, SAPK/JNK) do not indicate any changes in hepatic insulin receptor function in 3 month CBD-exposed male offspring, the decrease in hepatic Glut2 protein levels indicates that the liver may be one peripheral contributor to the glucose intolerance observed (Seyer *et al.* 2013, Thorens 2015). Specifically, a loss of hepatic Glut2 has been demonstrated not only to lead to reduced glucose uptake but also long-term glucose intolerance due to suppressed glucose-stimulated insulin secretion (GSIS) (Seyer *et al.* 2013). As such, future investigations should focus on defining the insulin response to glucose uptake in CBD-exposed offspring.

As previously discussed, we confirmed the presence of CBD within the livers of newborn pups. Given the association of the ECS with hepatic development and glucose metabolism, and the contributions of the liver to systemic glucose homeostasis (Han *et al.* 2016, Liu *et al.* 2016, Bazwinsky-Wutschke *et al.* 2019), we next assessed if there were long-lasting transcriptomic changes in the livers of 3-month-old male offspring exposed to 3 mg/kg of CBD during gestation. Specifically, we aimed to define any altered biological processes that could explain the hepatic contribution to the observed systemic glucose intolerance. Across the GO:BP, GO:MF, and KEGG databases, circadian rhythm terms were significantly enriched. In the liver, the circadian clock system synchronizes metabolism, nutrient uptake, and glucose homeostasis to the rhythm of the day–night cycle (Reinke & Asher 2016, Tahara & Shibata 2016). It is composed of the core clock genes and proteins, such as CLOCK, basic helix–loop–helix ARNT like 1 (BMAL1/ARNTL), period (PER), cryptochrome (CRY), and the nuclear receptors RAR-related orphan receptors (ROR), nuclear receptor subfamily 1 group D member 1 (REV-ERBA/NR1D1) and REV-ERBB/NR1D2, which are under the regulation of posttranscriptional and posttranslational modifications (Reinke & Asher 2016, Tahara & Shibata 2016). The core clock proteins regulate the cyclic expression of transcription factors and proteins involved in glucose metabolism such as KLF10, cyclic-AMP response element-binding protein (CREB), objective nuclear factor interleukin-3 regulated (NFIL3), and ubiquitin-specific protease 2 (USP2) (Guillaumond *et al.* 2010, Zhang *et al.* 2010, Molusky *et al.* 2012, Reinke & Asher 2016, Kang *et al.* 2017, Yuan *et al.* 2020).

Both endogenous cannabinoid and cannabinoid receptor expression are also regulated in the liver by the circadian clock (Bazwinsky-Wutschke *et al.* 2017, Sládek *et al.* 2019). Interestingly, circadian rhythm has been demonstrated to be dysregulated as a result of CBD exposure in murine microglial cells (Lafaye *et al.* 2019). This interaction between CBD and the hepatic circadian clock during development could lead to the sustained altered expression of circadian genes observed in the adult liver. However, whether CBD elicits the same effects in the developing liver as in microglial cells is yet unknown. There is strong evidence that alterations to the hepatic circadian clock increase the risk of developing nonalcoholic liver disease, obesity, and metabolic defects (Molusky *et al.* 2012, Reinke & Asher 2016, Mayeuf-Louchart *et al.* 2017, Shi *et al.* 2019). Furthermore, changes in the expression and function of circadian rhythm genes in the liver have been associated with glucose intolerance (Rudic *et al.* 2004, Turek *et al.* 2005, Molusky *et al.* 2012, Kang *et al.* 2017). Here, we observed a downregulation of *Nfil3* and an upregulation of *Usp2* in CBD-exposed male livers. *Nfil3* is a known negative regulator of gluconeogenesis (Kang *et al.* 2017), while *Usp2* stimulates glucose production (Molusky *et al.* 2012). Together, the changes in their respective expression levels lead to increased gluconeogenesis, which could contribute to the glucose intolerance observed. Our results and the literature suggest that altered expression of circadian rhythm genes in the liver of CBD-exposed 3-month-old male offspring could, in part, underlie the observed glucose intolerance. However, it is important to note that in the current study, the livers were collected at the same time in the day. Future studies should investigate if similar alterations to glucose tolerance and expression levels of circadian clock genes are observed throughout the day/night cycle.

In addition to circadian rhythm, functional enrichment analysis using the GO:BP database indicated that regulation of cellular and metabolic processes (Fig. 8A), cellular signaling and response to stimuli, developmental and morphogenic processes, and protein phosphorylation and phosphorous/phosphate containing compound metabolism were significantly enriched in CBD-exposed livers. This is concerning given that while the CBD insult occurred during gestation, these changes are detected to persist into adult life. To our knowledge, there have been no studies investigating the effects of perinatal CBD exposure on the developing liver. As such, the specific mechanisms underlying these changes are not yet understood. It is noteworthy that modulation of the ECS (e.g. due to the interaction with CBD) during development, has been observed to lead to developmental and metabolic deficits in a zebrafish model (Liu *et al.* 2016). Liu *et al.* demonstrated that pharmacological or genetic inhibition of cannabinoid receptor activity during zebrafish development resulted in deficits in liver development, structure, and metabolism (Liu *et al.* 2016). Specifically, the disrupted ECS led to livers with greater immature hepatocytes, altered morphology, impaired biliary tree formation, and increased steatosis. While we found no changes in hepatic triglyceride and cholesterol levels in 3-month-old vehicle-exposed and CBD-exposed offspring (Supplementary Fig. 2), it is very plausible that the dysglycemia might manifest later in life, similar to THC-exposed male offspring (Oke *et al.* 2020). Given we observed alterations in gene expression of developmental and morphogenic related genes, it will be of interest in future studies to examine if CBD-exposed livers also exhibit altered hepatocyte structure and biliary tree formation.

In summary, we have demonstrated for the first time that exposure CBD during gestation leads to glucose intolerance specifically in adult male offspring. This occurred without changes to the endocrine pancreas or fasting insulin levels. We postulate that altered gene expression of circadian rhythm clock machinery in the liver of the exposed males underlies, in part, the hepatic contribution to the overall glucose intolerance observed. Indeed, altered hepatic circadian rhythm is likely a key driver given its links to glucose intolerance (Rudic *et al.* 2004, Turek *et al.* 2005, Molusky *et al.* 2012, Kang *et al.* 2017). Transcriptomic analysis also discovered altered developmental and metabolic processes, suggesting for the first time that CBD negatively impacts fetal liver development and function. Future long-term studies will be important in assessing if the observed hepatic deficits culminate into hepatic pathologies (i.e. NAFLD) later in life. Collectively, these results indicate that despite its increased popularity, exposure to CBD alone in pregnancy may be detrimental to the metabolic health of the offspring. Moreover, the outcomes of these preclinical studies are imperative for clinicians and worldwide regulatory agencies in assessing the safety and efficacy of CBD use during human pregnancy.

## Supplementary Materials

Supplementary Material

Supplementary Table 1

Supplementary Table 2

## Declaration of interest

We do not have any conflicts of interest to disclose.

## Funding

This work was financially supported by IRF 2020–2021 grant from the Lawson Research Institute, London, Ontario, Canada to EJJA, and the Canadian Institutes of Health Researchhttp://dx.doi.org/10.13039/501100000024 Project Grant (R4228A28) and a Canadian Heart and Stroke Foundationhttp://dx.doi.org/10.13039/100013751 Grant-in-Aid (G-19-0026343) to DBH. SV was also supported by an OGGS and OGS scholarship.
