# Supplementary material for: Gestational exposure to cannabidiol leads to glucose intolerance in 3-month-old male offspring
Source: J Endocrinol. 2023 Nov 23;260(1):e230173. doi: 10.1530/JOE-23-0173 (PMC10762538; doi:10.1530/JOE-23-0173)
Supplement: Supplementary Material [file supplementary_material.pdf]

Gestational exposure to cannabidiol leads to glucose intolerance in 3-month-old male offspring.

Sebastian R. Vanin, Kendrick Lee, Mina Nashed, Brennan Tse, Mohammed Sarikahya, Sukham Brar, Gregg Tomy, Amica-Mariae Lucas, Thane Tomy, Steven Laviolette, Edith Arany, and Daniel B. Hardy

SUPPLEMENTARY INFORMATION

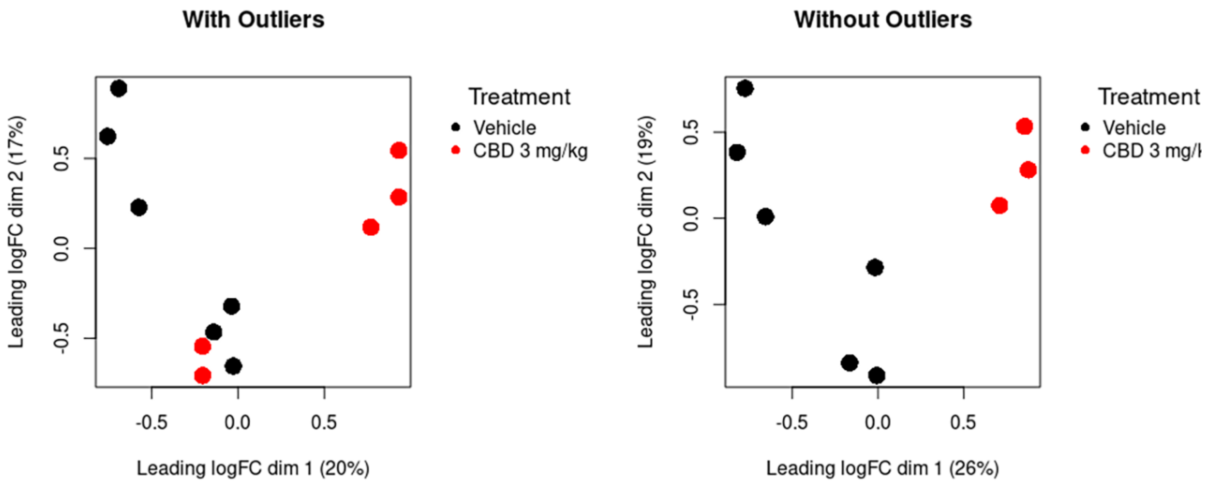

Supplemental Figure 1: Principal Component Analysis Plot of 3-month Liver from Vehicle and CBD-Exposed Offspring.

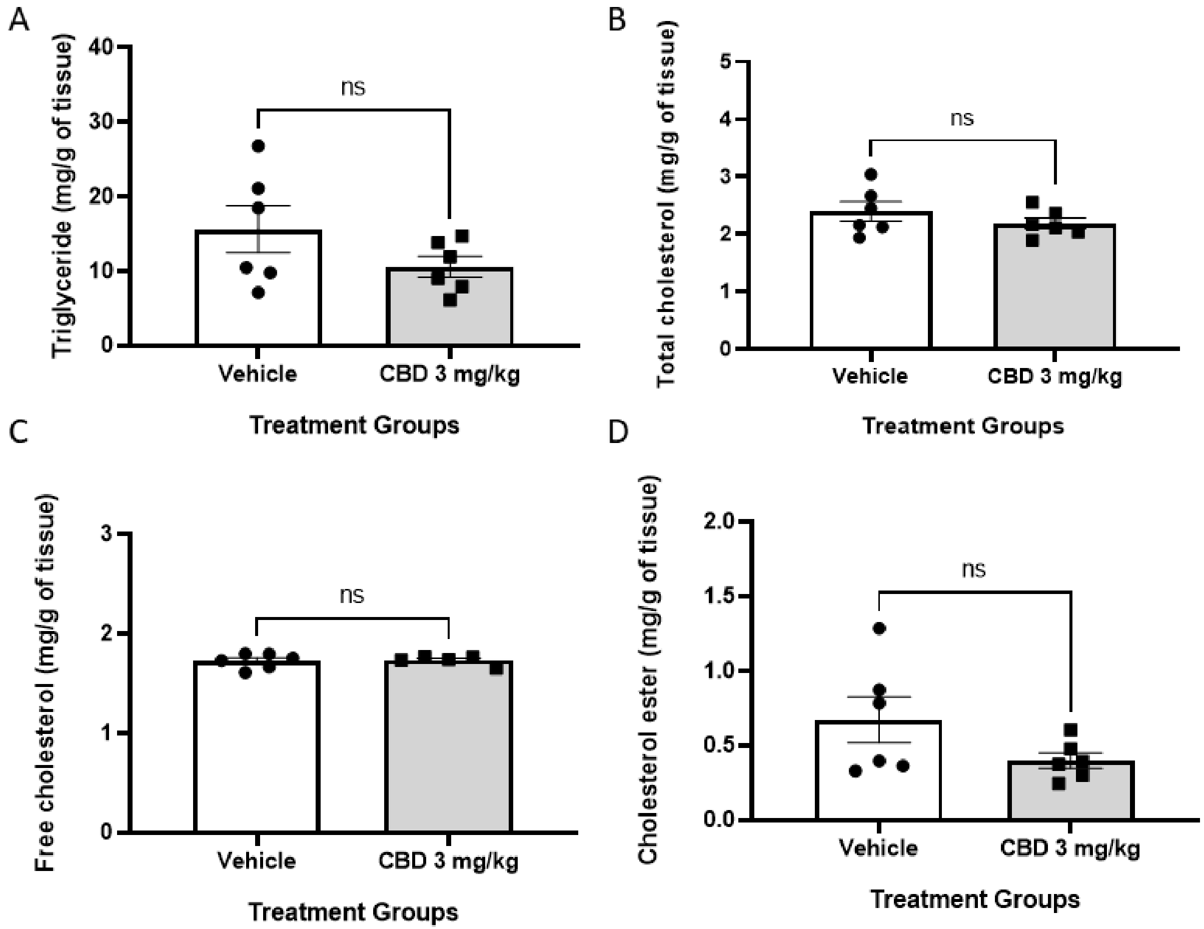

Supplemental Figure 2: The effects of gestational CBD exposure on cholesterol and triglyceride levels in the liver of exposed male offspring at three months of age. At three months of age, (A) hepatic triglycerides (B) total cholesterol, (C) free cholesterol, and (D) cholesterol esters were assessed in both vehicle and CBD-exposed (mg of lipid/g of tissue). Data are expressed as the mean ± SEM. Significant differences in were assessed by a Student's unpaired t-test (*, $p < 0.05$). Circulating and hepatic triglyceride and cholesterol measurements were detected using the Cobas® Mira S analyzer as previously published. For triglyceride measurements, triglycerides were hydrolyzed by lipoprotein lipase to glycerol and fatty acids. Glycerol was then phosphorylated to glycerol-3-phosphate by ATP in a reaction catalyzed by glycerol kinase (GK). The oxidation of glycerol-3-phosphate was catalyzed by glycerol phosphate oxidase (GPO) to form dihydroxyacetone phosphate and hydrogen peroxide (H2O2). In the presence of peroxidase, H2O2 alters the oxidative coupling of 4-chlorophenol and 4-aminophenazone to form a red-colored quinoneimine dye, which was measured at 512 nm. The increase in absorbance is directly proportional to the concentration of triglycerides in the sample. For cholesterol measurements, cholesterol esterase cleaved cholesterol esters, which then were converted to choleste-4-en-3-one and H2O2 by cholesterol oxidase. Cholesterol levels were quantified using a colorimetric assay that measured the breakdown of H2O2 via the Trinder reaction as previously described.
